# Supplementary material for: Characterizing the Dynamic Protein and Amino Acid Deposition in Tissues of Pregnant Gilts: Implications for Stage-Specific Nutritional Strategies
Source: Animals (Basel). 2025 Jul 18;15(14):2126. doi: 10.3390/ani15142126 (PMC12291760; doi:10.3390/ani15142126)
Supplement: Supplementary file 1 [file animals-15-02126-s001.zip › animals-3624466-supplementary.pdf]

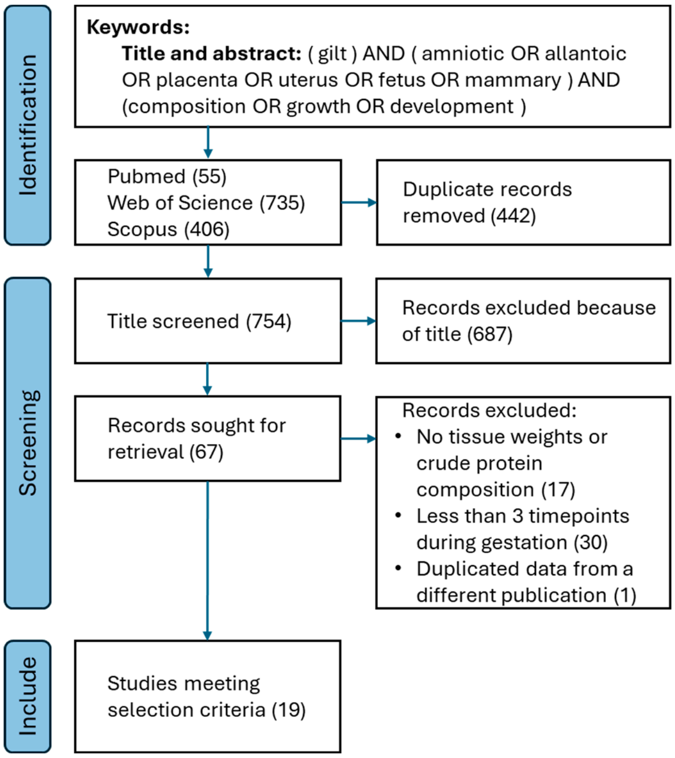

Figure S1. Flow diagram indicating the number of citations and publications included and excluded in each level of the systematized literature search on the tissue wet weight and crude protein composition in gestating gilts, adapted from PRISMA guidelines [5].

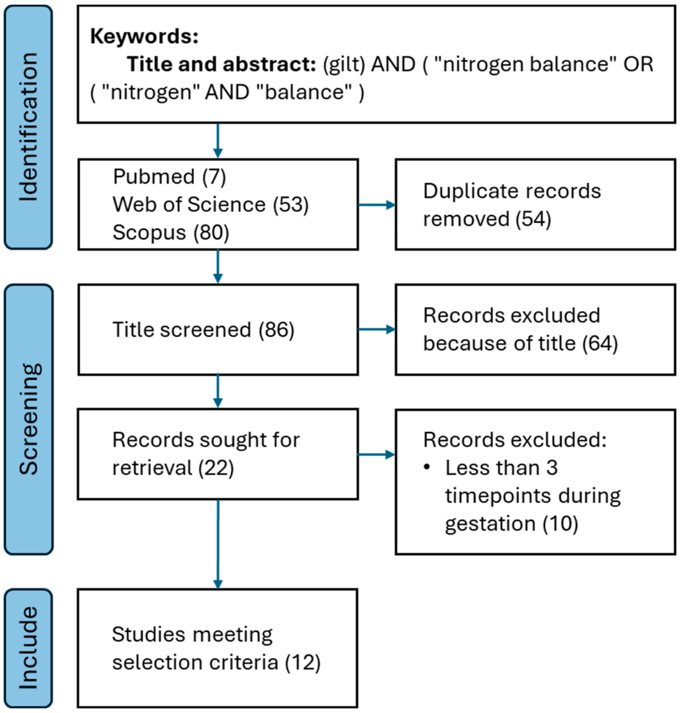

Figure S2. Flow diagram indicating the number of citations and publications included and excluded in each level of the systematized literature search on nitrogen retention during gestation in gilts, adapted from PRISMA guidelines [5].

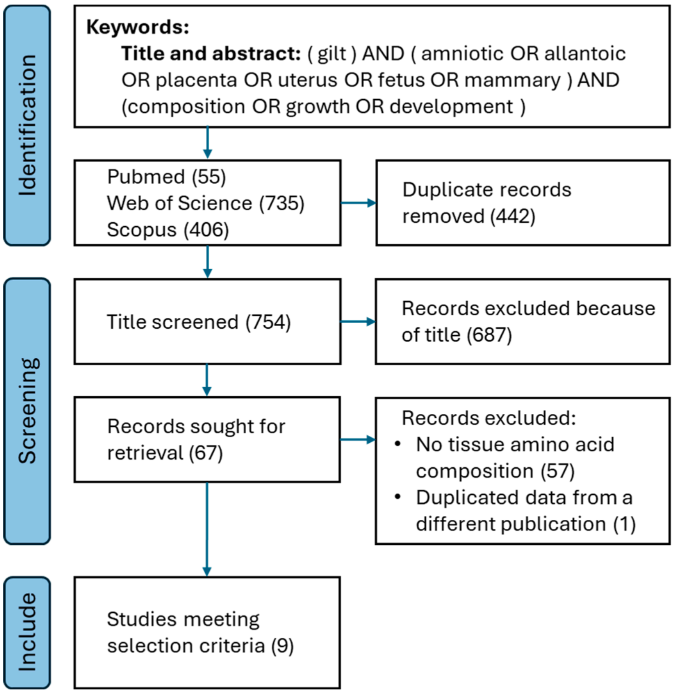

Figure S3. Flow diagram indicating the number of citations and publications included and excluded in each level of the systematized literature search on tissue amino acid composition during gestation in gilts, adapted from PRISMA guidelines [5].

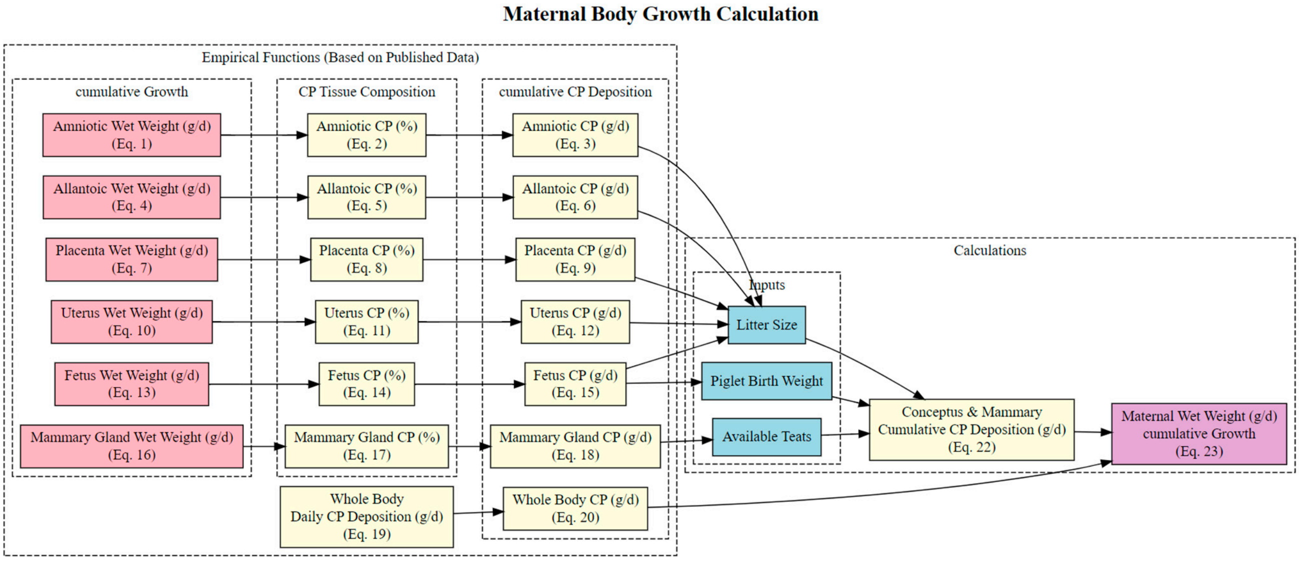

Figure S4. Diagram illustrating how the growth model integrates equations 1 through 22 with the objective of estimating the maternal body wet weight.

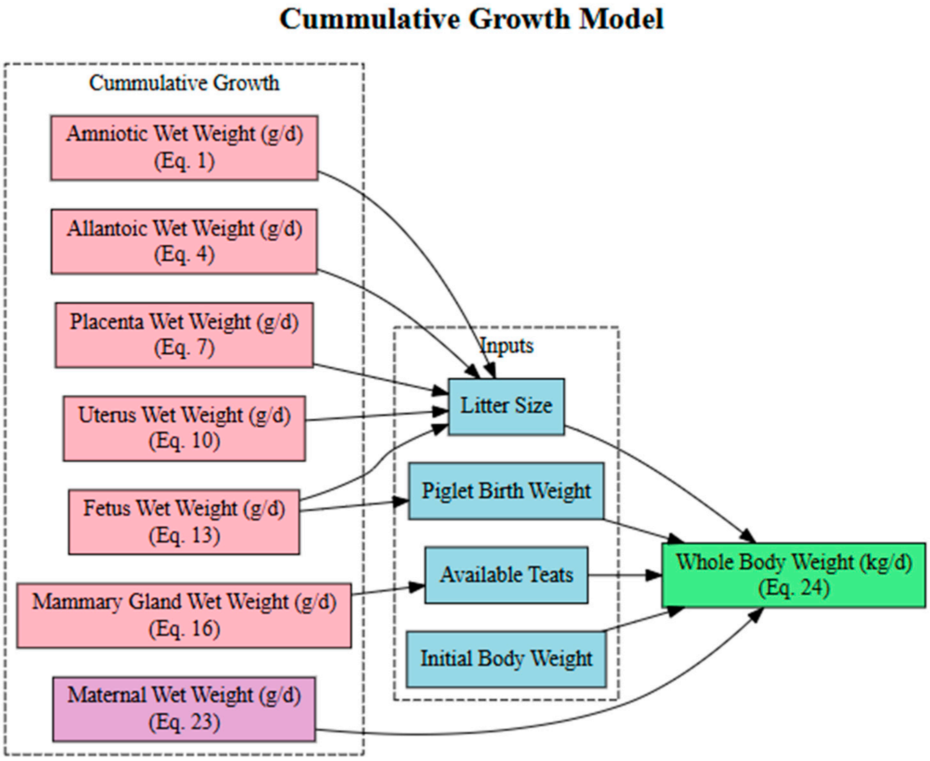

Figure S5. Diagram illustrating how the growth model calculates the whole-body weight gain during gestation by integrating the calculated wet weights of the products of conception and the mammary gland with the calculated maternal body weight.

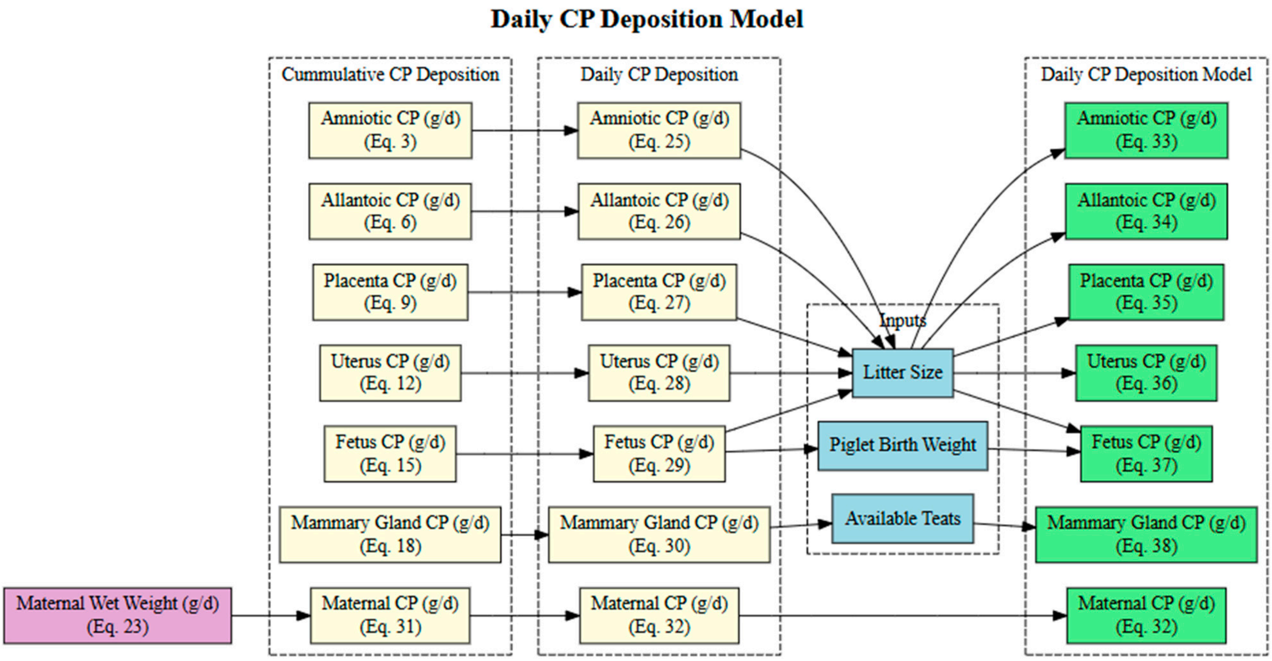

Figure S6. Diagram illustrating how the crude protein deposition model incorporates all the required equations and performs its calculations.

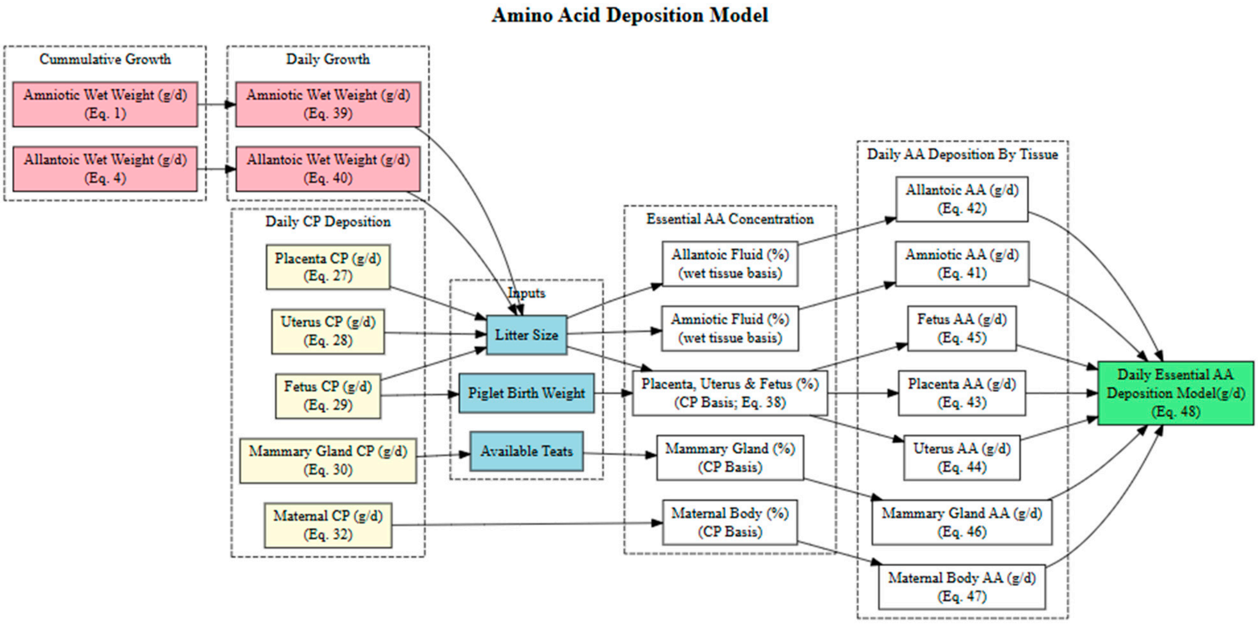

Figure S7. Diagram illustrating how the amino acid deposition model incorporates all the required equations and performs its calculations.

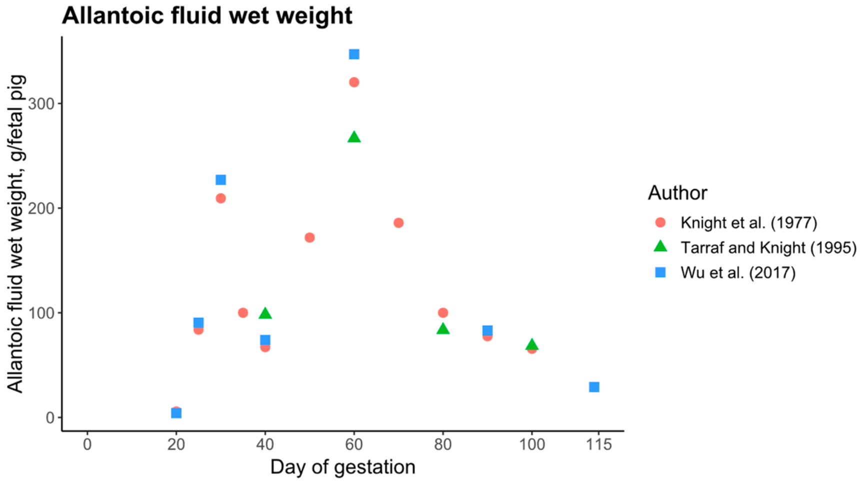

Figure S8. Allantoic fluid wet weight across gestation in sows, based on a systematized literature search of studies reporting data at more than three time points during gestation. Data extracted from Knight et al. (1977) [12], Tarraf and Knight (1995) [13], and Wu et al. (2017) [14].

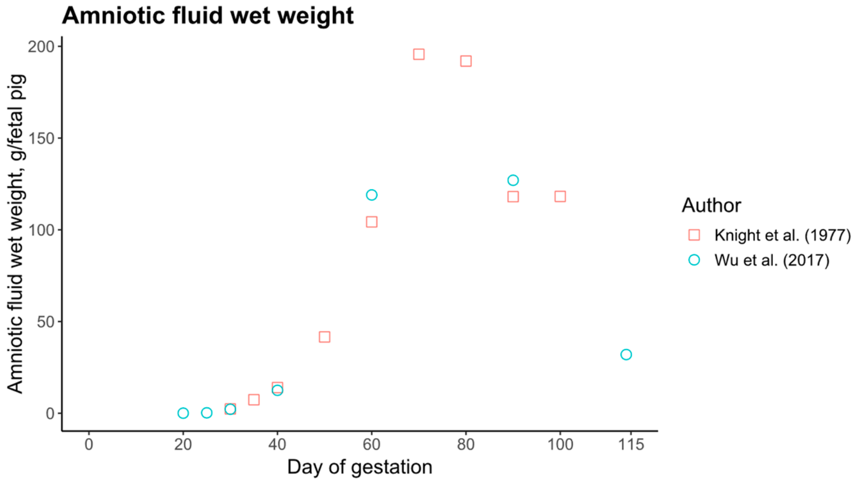

Figure S9. Amniotic fluid wet weight across gestation in sows, based on a systematized literature search of studies reporting data at more than three time points during gestation. Data extracted from Knight et al. (1977) [12] and Wu et al. (2017) [14].

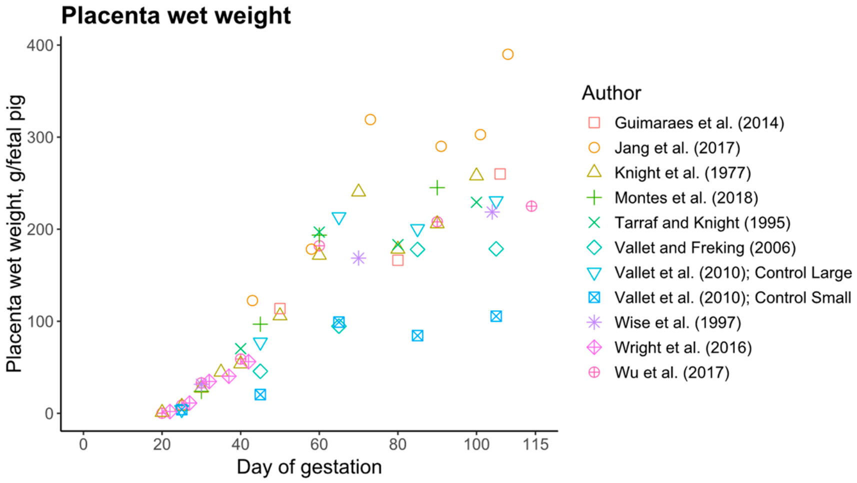

Figure S10. Placenta wet weight across gestation in sows, based on a systematized literature search of studies reporting data at more than three time points during gestation. Data extracted from Guimarães et al. (2014) [15], Jang et al. (2017) [3], Knight et al. (1977) [12], Montes et al. (2018) [17], Tarraf and Knight (1995) [13], Vallet and Freking (2006) [18], Vallet et al. (2010) [19], Wise et al. (1997) [20], Wright et al. (2016) [21], and Wu et al. (2017) [14].

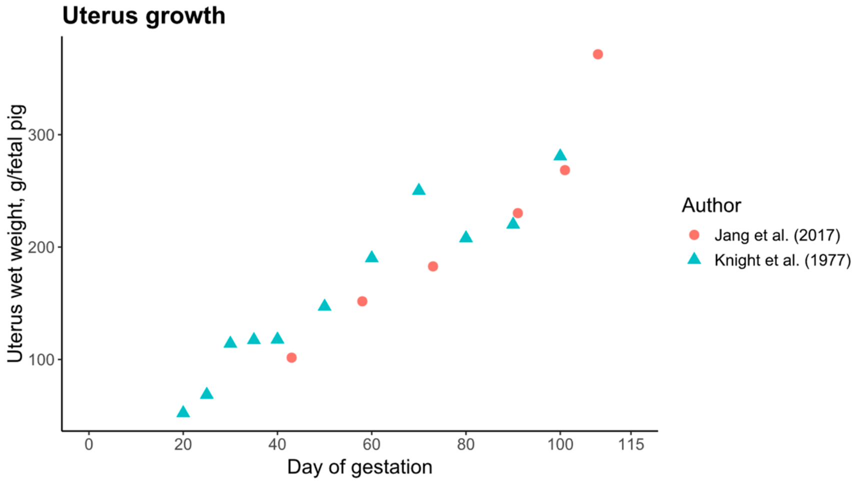

Figure S11. Uterus wet weight across gestation in sows, based on a systematized literature search of studies reporting data at more than three time points during gestation. Data extracted from Jang et al. (2017) [3] and Knight et al. (1977) [12].

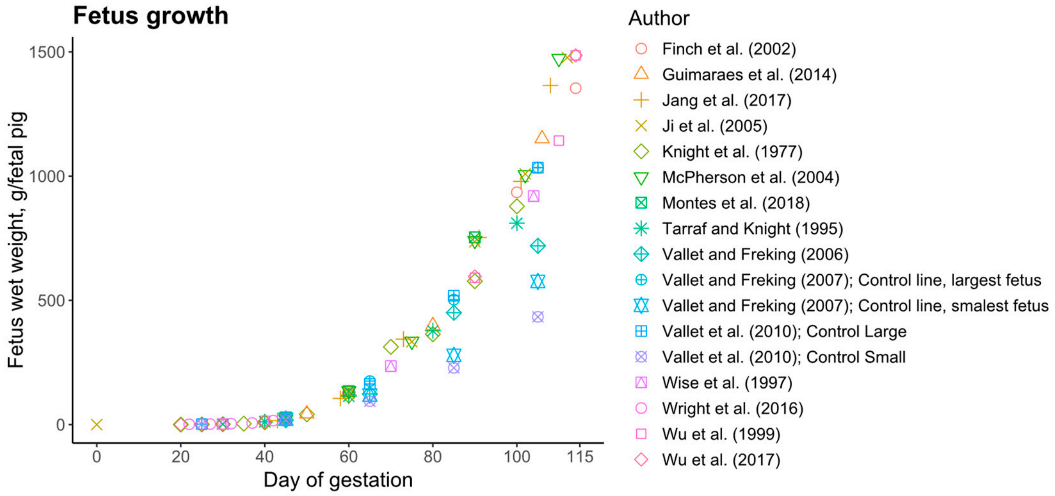

Figure S12. Fetus wet weight across gestation in sows, based on a systematized literature search of studies reporting data at more than three time points during gestation. Data extracted from Finch et al. (2002) [22], Guimarães et al. (2014) [15], Jang et al. (2017) [3], Ji et al. (2005) [2], Knight et al. (1977) [12], McPherson et al. (2004) [16], Montes et al. (2018) [17], Tarraf and Knight (1995) [13], Vallet and Freking (2006) [18], Vallet and Freking (2007) [23], Vallet et al. (2010) [19], Wise et al. (1997) [20], Wright et al. (2016) [21], Wu et al. (1999) [4], and Wu et al. (2017) [14].

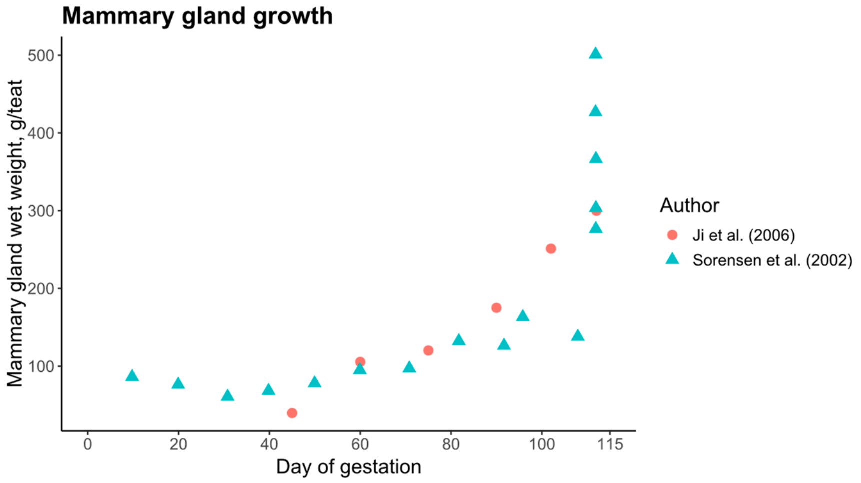

Figure S13. Mammary gland wet weight across gestation in sows, based on a systematized literature search of studies reporting data at more than three time points during gestation. Data extracted from Ji et al. (2006) [24] and Sørensen et al. (2002) [25].

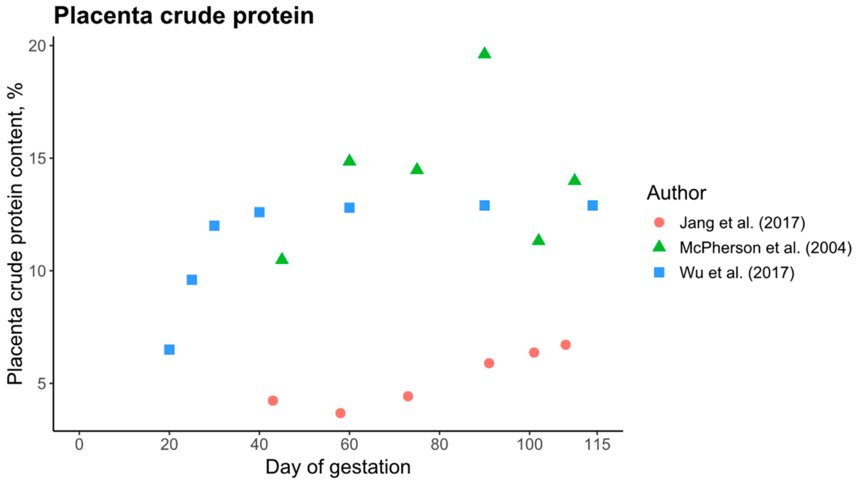

Figure S14. Placenta crude protein content across gestation in sows, based on a systematized literature search of studies reporting data at more than three time points during gestation. Data extracted from Jang et al. (2017) [3], McPherson et al. (2004) [16], and Wu et al. (2017) [14].

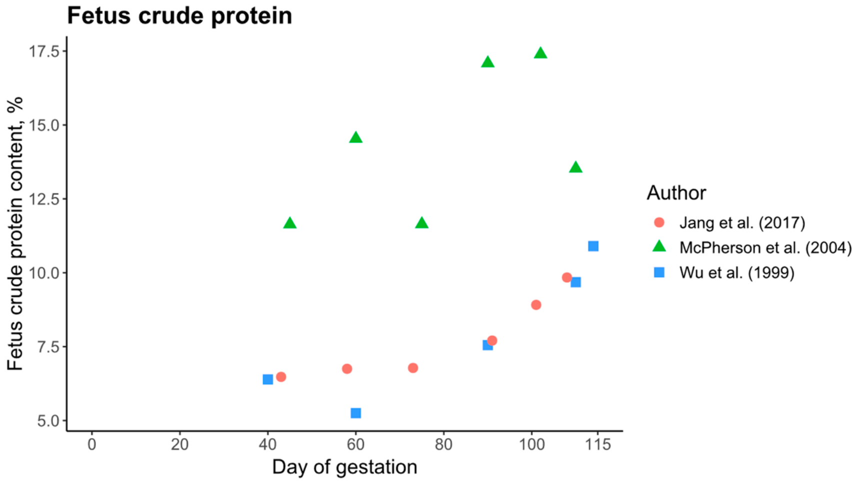

Figure S15. Fetus crude protein content across gestation in sows, based on a systematized literature search of studies reporting data at more than three time points during gestation. Data extracted from Jang et al. (2017) [3], McPherson et al. (2004) [16], and Wu et al. (1999) [4].

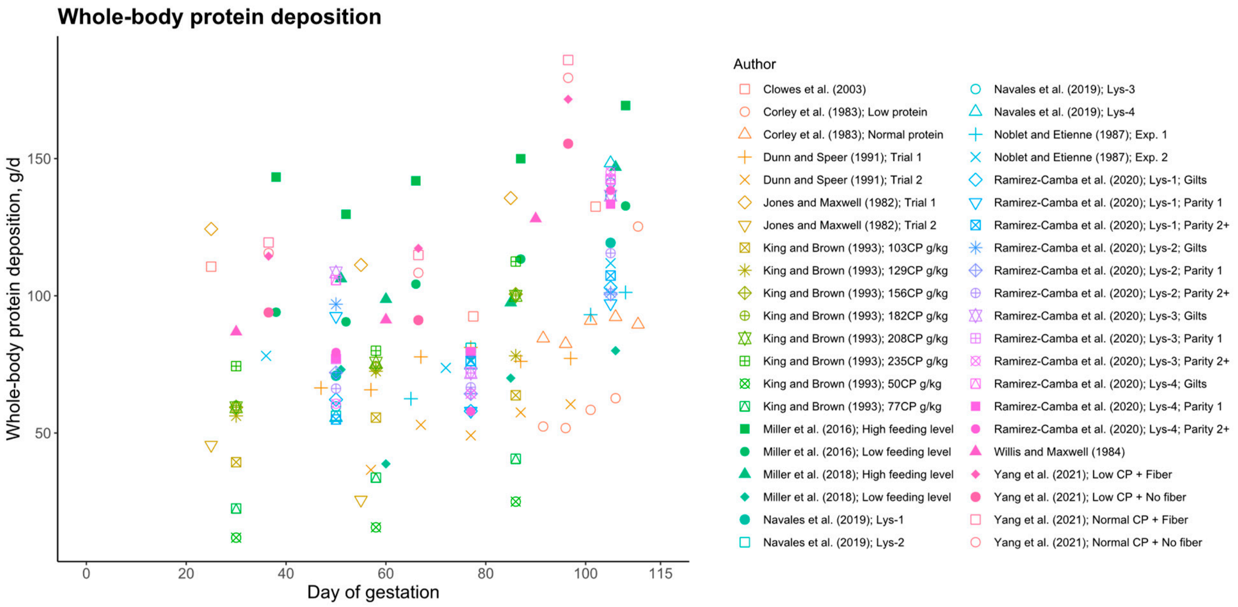

Figure S16. Whole-body protein deposition data across gestation in sows, compiled from a systematized literature search of studies reporting more than three time points during gestation. One protein deposition value reported in Jones and Maxwell (1982) was negative and was excluded from the analysis. Data extracted from Clowes et al. (2003) [26], Corley et al. (1983) [27], Dunn and Speer (1991) [28], Jones and Maxwell (1982) [29], King and Brown (1993) [30], Miller et al. (2016) [9], Miller et al. (2018) [31], Navales et al. (2019) [32], Noblet and Etienne (1987) [33], Ramirez-Camba et al. (2020) [34], Willis and Maxwell (1984) [35], and Yang et al. (2021) [36].

R Script S1: Code for reproducing the dynamic protein and amino acid deposition in tissues of pregnant gilts

Overview

This R script serves as supplementary information, presenting the calculations performed for estimating a model designed to study the growth, crude protein (CP), and amino acid (AA) deposition in the pregnant gilt. The detailed calculations are described in the accompanying manuscript, and this file provides the corresponding code for the equations used, the algorithm, and their implementation in the model. The intent of sharing this code is to promote transparency, encourage its use, and present a model that can be improved by other scientists as more information becomes available.

This model and its associated code are shared under the Creative Commons Attribution 4.0 International License (CC BY 4.0). This means the code can be reused, shared, and adapted, provided appropriate attribution is given to the original authors.

The gestating gilt model is comprised of three sub-models, which are detailed below:

1. *Growth model*

```
#Libraries used
library(ggplot2)
library(dplyr)
library(reshape2)
library(plotly)

### MODEL INPUTS ###
litter_size <- 13.5
piglet_bw <- 1.4
teats <- 14
initial_BW <- 150
days <- seq(1, 115, 1)

###CALCULATIONS ###

# Amniotic fluid
#Equation 1
Amniotic_wet_weight <- 321.22 / (1 + 2400 * exp(-0.118 * days)) - 196.86 / (1
+ (days / 81.4)^-27.83)
#Equation 2
Amniotic_CP <- (0.1928 + 0.00107 * days) / (1 - 0.0328 * days + 0.0002928 *
days^2)
#Equation 3
Amniotic_CP_total <- Amniotic_wet_weight* Amniotic_CP/100

# Allantoic fluid
#Equation 4
Allantoic_wet_weight <- exp(420.4 - 2774.5 / days - 94.91 * log(days)) +  1 /
(0.0823 - 0.002596 * days + 0.0000213 * days^2)
#Equation 5
Allantoic_CP <- exp(419.15 - 3595.38 / days - 89.56 * log(days)) +  1 /
(21.45 - 0.4739 * days + 0.002765 * days^2)
#Equation 6
Allantoic_CP_total <- Allantoic_wet_weight* Allantoic_CP/100

# Placenta
#Equation 7
Placenta_wet_weight <- 217.503984088964 / (1 + exp(5.89560473900821 -
0.124681756057846 * days))
#Equation 8
Placenta_CP <- 12.8081166572279 / (1 + exp(10.9428173490322 -
0.400479683307378 * days))^(1 / 4.39763962618055)
```

```
#Equation 9
Placenta_CP_total <- Placenta_wet_weight* Placenta_CP/100

# Uterus
#Equation 10
Uterus_wet_weight <- days / (0.451351807782315 - 0.0000000197994076812918 *
days^3.86501292501773)
#Equation 11
Uterus_CP <- 5.277 + 0.147 * days - (4.606 / (1 + 2093 * exp(-0.127 * days)))
- 0.598 * days^(0.0043 * days)
#Equation 12
Uterus_CP_total <- Uterus_wet_weight* Uterus_CP/100

# Fetus
#Equation 13
Fetus_wet_weight <- 0.0000163 * days^3.859
#Equation 14
Fetus_CP <- 9.286 + 0.194 * days - (6.027 / (1 + 817735 * exp(-0.24 * days)))
- 9.277 * days^(0.00088 * days)
#Equation 15
Fetus_CP_total <- Fetus_wet_weight* Fetus_CP/100

# Mammary gland
#Equation 16
Mammary_gland_wet_weight <- 0.01778995538930640 * days^2.06069502332356
#Equation 17
Mammary_gland_CP <- -14.3237419299505 * days / (-213.7371268178330 + days)
#Equation 18
Mammary_gland_CP_total <- Mammary_gland_wet_weight* Mammary_gland_CP/100

#Equation 19
Daily_whole_body_CP_retention <- function(day) {
  841.7 + 2.14 * day - 25.24 / (1 + 86700000 * exp(-0.39 * day)) - 771 *
(day^(0.0003 * day))
}

########### Growth model adjustments to fit empirical data (model
calibration) ###########
# In this section Equation 19 is replaced by Equation 50 which resulted from
a calibration process to match empirical data.

# Equation 50 = Equation 19 - Equation 49
Daily_whole_body_CP_retention_calibrated_eq50 <- function(day) {
    return(
        # Equation 19
        841.7 + 2.14 * day - 25.24 / (1 + 86700000 * exp(-0.39 * day)) - 771
* day^(0.0003 * day) -

        # Equation 49
        ((299.86 * day^(-7.20585)) / ((8.3295)^(-7.20585) + day^(-7.20585)))
    )
}

# Uncomment the following line to calculate the calibrated model by
incorporating Equation 50. If the line below is not uncommented the original
model is calculated using Equation 19

#Daily_whole_body_CP_retention<-Daily_whole_body_CP_retention_calibrated_eq50

##############################################################################
##############

########### CP and essential AA deposition models calibration ###########
# In this section Equation 19 is replaced by the Equation shown in Fig 14
which resulted from removing the assumption of reduced CP deposition during
early gestation when developing the whole boyd CP deposition from Miller et
al. (2016) data.

#Equation in Figure 14
Daily_whole_body_CP_retention_calibrated_fig14 <- function(day) {
```

```
  return(135.228098 - 0.853296* day + 0.009299* day^2)
}

# Uncomment the following line to calculate the model not considering the
assumption of reduced CP deposition during early gestation. If the line below
is not uncommented the original model is calculated using Equation 19.

#Daily_whole_body_CP_retention<-
Daily_whole_body_CP_retention_calibrated_fig14

##########################################################################

#Equation 20
cumulative_whole_body_CP_retention <- sapply(1:115, function(day)
integrate(Daily_whole_body_CP_retention, lower = 1, upper = day)$value)

#Equation 22
Conceptus_n_Mammary_CP <- litter_size * (Amniotic_CP_total +
Allantoic_CP_total +
  Placenta_CP_total + Uterus_CP_total + Fetus_CP_total * (piglet_bw /
1.46023)) +
  teats * Mammary_gland_CP_total

#Equation 23
Maternal_wet_weight <- ((cumulative_whole_body_CP_retention -
Conceptus_n_Mammary_CP) / 1000) / 0.22

#Equation 24
WB_wet <-((Amniotic_wet_weight + Allantoic_wet_weight + Placenta_wet_weight +
Uterus_wet_weight +
               Fetus_wet_weight * (piglet_bw / 1.46023)) * litter_size +
Mammary_gland_wet_weight * teats)/1000 + Maternal_wet_weight + initial_BW

### PLOT GROWTH MODEL ###

#Data frame for plot
WB_wet_data <- data.frame(day = days,
                     y = WB_wet,
                     Eq = "Equation 24")

# ggplot for the growth model
Plot1 <- ggplot(WB_wet_data,aes(x = day, y = y,linetype=Eq)) +
  geom_line() +
  scale_x_continuous(breaks = c(seq(0, 100, 20), 115), limits = c(0, 115)) +
  theme_classic() +
  labs(shape = NULL, linetype = NULL) +
  xlab("Day of gestation")+
  ylab("Whole body weight, kg")+
  theme(legend.position = c(0.2, 0.85))

ggplotly(Plot1)
```

1.1 Growth model by tissue type

```
### The code used for plotting the growth model by tissue type is not
described in the associated manuscript but is shown below ###
WB_wet_by_tissue <- data.frame(
  day = days,
  Amniotic = litter_size * Amniotic_wet_weight / 1000,
  Allantoic = litter_size * Allantoic_wet_weight / 1000,
  Placenta = litter_size * Placenta_wet_weight / 1000,
  Uterus = litter_size * Uterus_wet_weight / 1000,
  Fetus = litter_size * Fetus_wet_weight * (piglet_bw / 1.46023) / 1000,
  Mammary = teats * Mammary_gland_wet_weight / 1000,
  Maternal = Maternal_wet_weight
)
# Convert data to long format for plotting
WB_long <- melt(WB_wet_by_tissue, id.vars = "day")
```

```
# Adjust variable names for better presentation
WB_long$variable <- gsub("Maternal", "Maternal body", WB_long$variable)
WB_long$variable <- gsub("Amniotic", "Amniotic fluid", WB_long$variable)
WB_long$variable <- gsub("Allantoic", "Allantoic fluid", WB_long$variable)
WB_long$variable <- gsub("Mammary", "Mammary gland", WB_long$variable)

#Order the tissues according to the desired display order in the plot.
Levels <- c("Maternal body", "Fetus", "Placenta", "Uterus", "Mammary gland",
"Amniotic fluid", "Allantoic fluid")
WB_long$variable<-factor(WB_long$variable, levels=Levels)

#Set up the Color for each tissue
Col<-c('#7CCBB3', #Maternal body
    "#1f77b4", #Fetus
    '#E788C3', #Placenta
    '#8FA0CC', #Uterus
    '#FFDD47', #Mammary gland
    '#9467bd', #Amniotic fluid
    '#7f7f7f' #Allantoicfluid
  )
#variable f refers to font characteristics
f <- list(family = "Helvetica", size = 14, color = "black")
#variable x refers to x axis label and font
x <- list(title = "Day of gestation", font=f)
#variable x refers to y axis label and font
y <- list(title = "Cummulative weight gain, kg", font=f)

# Create interactive plot
Plot2 <- plot_ly( WB_long,
              type = 'scatter',
              alpha = 0.5,
              alpha_stroke = 1,
              colors = Col,
              x = ~day, y = ~value, color = ~variable,
              mode = 'line',
              fill = 'tonexty',
              stackgroup = 'one',
              line = list(width = 1)) %>%
  layout( xaxis = x,
    yaxis = y,
    font = f,
    margin = list(l = 50, r = 50, b = 80, t = 50),  # Adjust margins as
needed
    legend = list(
      orientation = 'h',  # Horizontal legend
      x = 0.5,  # Center the legend
      xanchor = 'center',
      y = 1.1  # Position the legend at the top
    )
  ) %>%
  config(displaylogo = FALSE,displayModeBar = FALSE)

# Display the plot
Plot2
```

2. *Daily whole-body crude protein deposition model*

```
#The function diff() can be used to estimate the discrete derivative
#Equation 25
Amniotic_CP_daily <- diff(Amniotic_CP_total)
#Equation 26
Allantoic_CP_daily <- diff(Allantoic_CP_total )
#Equation 27
Placenta_CP_daily <- diff(Placenta_CP_total)
#Equation 28
Uterus_CP_daily <- diff(Uterus_CP_total)
#Equation 29
Fetus_CP_daily <- diff(Fetus_CP_total)
#Equation 30
```

```
Mammary_gland_CP_daily <- diff(Mammary_gland_CP_total)
#Equation 31
Maternal_CP <- Maternal_wet_weight * 1000 * 0.22
#Equation 32
Maternal_CP_daily <- diff(Maternal_CP)

#Equation 33
Amniotic_CP_daily_total <- Amniotic_CP_daily * litter_size
#Equation 34
Allantoic_CP_daily_total <- Allantoic_CP_daily * litter_size
#Equation 35
Placenta_CP_daily_total <- Placenta_CP_daily * litter_size
#Equation 36
Uterus_CP_daily_total <- Uterus_CP_daily * litter_size
#Equation 37
Fetus_CP_daily_total <- Fetus_CP_daily * litter_size * (piglet_bw / 1.46023)
#Equation 38
Mammary_gland_CP_daily_total <- Mammary_gland_CP_daily * teats

# Combine daily CP deposition into a data frame for data visualization
Daily_CP_by_tissue <- data.frame(
  day = seq(1, 114, 1),  # Days 1 to 114
  Amniotic = Amniotic_CP_daily_total,
  Allantoic = Allantoic_CP_daily_total,
  Placenta = Placenta_CP_daily_total,
  Uterus = Uterus_CP_daily_total,
  Fetus = Fetus_CP_daily_total,
  Mammary = Mammary_gland_CP_daily_total,
  Maternal = Maternal_CP_daily
)

# Convert data to long format for visualization
CP_long <- melt(Daily_CP_by_tissue, id.vars = "day")
CP_long$variable <- gsub("Maternal", "Maternal body", CP_long$variable)
CP_long$variable <- gsub("Amniotic", "Amniotic fluid", CP_long$variable)
CP_long$variable <- gsub("Allantoic", "Allantoic fluid", CP_long$variable)
CP_long$variable <- gsub("Mammary", "Mammary gland", CP_long$variable)

#Order the tissues according to the desired display order in the plot.
Levels <- c("Fetus", "Placenta", "Uterus", "Mammary gland", "Amniotic fluid",
            "Allantoic fluid", "Maternal body")

CP_long$variable <- factor(CP_long$variable, levels = Levels)

# Define color palette
Col <- c(
  "#1f77b4", # Fetus
  '#E788C3', # Placenta
  '#8FA0CC', # Uterus
  '#FFDD47', # Mammary gland
  '#9467bd', # Amniotic fluid
  '#7f7f7f', # Allantoic fluid
  '#7CCBB3'  # Maternal body
)

# Plot settings
f <- list(family = "Helvetica", size = 14, color = "black")
x <- list(title = "Day of gestation", font = f)
y <- list(title = "Crude protein deposition, g/d", range = c(0, 162), font =
f)

# Create interactive plot using the plotly library
Plot3 <- plot_ly(
  CP_long,
  type = 'scatter',
  alpha = 0.5,
  alpha_stroke = 1,
  colors = Col,
  x = ~day, y = ~value, color = ~variable,
  mode = 'line',
```

```
  fill = 'tonexty',
  stackgroup = 'one',
  line = list(width = 1)
) %>%
  layout( xaxis = x, yaxis = y, font = f,
    margin = list(l = 50, r = 50, b = 80, t = 50),
    legend = list( orientation = 'h', x = 0.5,
      xanchor = 'center',
      y = 1.1
    )
  ) %>%
  config(displaylogo = FALSE, displayModeBar = FALSE)

# Display the plot
Plot3
```

3. Daily whole-body amino acid deposition model

3.1. Daily AA deposition calculated for amniotic fluid

```
#Equation #39
Amniotic_daily_AA <- diff(Amniotic_wet_weight)

#Essential AA concentration needed for Equation 42
Amniotic_AA <- data.frame(
  Lys = 0.04872,
  Arg = 0.03567,
  His = 0.00850,
  Ile = 0.01305,
  Leu = 0.01220,
  Met = 0.00761,
  Cys = 0.00448,
  Phe = 0.00797,
  Thr = 0.01540,
  Trp = 0.00368,
  Val = 0.02542
)
# Create a data frame to store results from Equation 41
Amniotic_daily_AA_composition<-data.frame(day=seq(1,114,1))

#Equation 41 calculated iterative for each essential amino acid
for (AA in names(Amniotic_AA)) {
  Amniotic_daily_AA_composition[[AA]] <- Amniotic_daily_AA * litter_size * (Am
niotic_AA[[AA]] / 1000)
}

# Display the calculated values for all essential AAs by day of gestation
head(Amniotic_daily_AA_composition)
```

3.2. Daily AA deposition calculated for allantoic fluid

```
#Equation #40
Allantoic_daily_AA <- diff(Allantoic_wet_weight)

#Essential AA concentration needed for Equation 42
Allantoic_AA <- data.frame(
  Lys = 0.25291,
  Arg = 0.41420,
  His = 0.05768,
  Ile = 0.01108,
  Leu = 0.01174,
  Met = 0.00642,
  Cys = 0.02423,
  Phe = 0.00764,
  Thr = 0.09979,
  Trp = 0.01317,
  Val = 0.03028
)
```

```r
# Create a data frame to store results from Equation 42
Allantoic_daily_AA_composition<-data.frame(day=seq(1,114,1))

#Equation 42 calculated iterative for each essential amino acid
for (AA in names(Allantoic_AA)) {
  Allantoic_daily_AA_composition[[AA]] <- Allantoic_daily_AA * litter_size *
(Allantoic_AA[[AA]] / 1000)
}

# Display the calculated values for all essential AAs by day of gestation
head(Allantoic_daily_AA_composition)
```

3.3. Daily AA deposition calculated for placenta

```
#Coefficients for constructing quadratic functions representing essential AA
concentrations in the placenta (Table 4; columns 1, 2, 3, and 4)
Placenta_coefficients <- data.frame(
  AminoAcid = c("Lys", "Arg", "His", "Ile", "Leu", "Met", "Cys", "Phe", "Thr",
"Trp", "Val"),
  Intercept = c(6.39227250408641, 8.6725564458959, 1.82439866328083,
4.56139980597124, 8.24275603405756, 1.99636119602192, 1.00338570936719,
5.28234967096861, 3.87021486830426, 1.16171155578071, 5.83239125817669),
  Linear = c(0.00857506284582966, -0.0338710102592167, 0.00554800881948121, -
0.0186896256392842, -0.0243216188775185, 0.00148306821094742,
0.00328983710166131, -0.010150957165451, 0.00874557402017799,
             -0.00333733259275287,-0.0118911889855624),
  Quadratic = c(-0.000090082739030308, 0.000224220535061544, -
0.000029839356987675, 0.0000935760922890758, 0.000123655383692164, -
0.000015558591265459, -0.000216267561780224, 0.0000440192984984829, -
0.0000672878810027029,
                0.0000244138555397896, 0.0000592929049038421)
  )

# Prepare a data frame to store the results of Equation 43
Placenta_daily_AA_composition <- data.frame(day = days)

#Equation 43 calculated iterative for each essential amino acid
for (i in 1:nrow(Placenta_coefficients)) {
  #Coefficients needed to calculate Eq. 38 for each essential AA
  amino_acid <- Placenta_coefficients$AminoAcid[i]
  intercept <- Placenta_coefficients$Intercept[i]
  linear <- Placenta_coefficients$Linear[i]
  quadratic <- Placenta_coefficients$Quadratic[i]

  # Calculate the quadratic function representing the each essential AA (Eq.
38) for  placenta
  Eq_38_placenta <- intercept + linear * Placenta_daily_AA_composition$day +
quadratic * Placenta_daily_AA_composition$day^2

  #Equation 43
  Placenta_daily_AA_composition[[amino_acid]] <- Placenta_CP_daily *
litter_size * Eq_38_placenta/100
}

# Display the calculated values for all essential AAs by day of gestation
head(Placenta_daily_AA_composition)
```

3.4. Daily AA deposition calculated for uterus

```
#Coefficients for constructing quadratic functions representing essential AA
concentrations in the uterus (Table 4; columns 1, 5, 6, and 7)
Uterus_coefficients <- data.frame(
  AminoAcid = c("Lys", "Arg", "His", "Ile", "Leu", "Met", "Cys", "Phe", "Thr",
"Trp", "Val"),
  Intercept = c(8.1298880922738, 8.51935160207145, 3.21434078612399,
4.21292626491918, 9.26434116078136, 1.72059210266652, 2.59779726947862,
5.35862127082234, 4.99689358818043, 1.11894494736435, 6.01331351868042),
  Linear = c(-0.0387332821098945, -0.0405744556432403, -0.0229908684217657, -
0.020150667640434, -0.0432956493413511, -0.00155417874744175, -
0.0228314982910052, -0.0304409722787905, -0.0249214677194754,
             -0.00312091264295533, -0.0278146162698911),
  Quadratic = c(0.000263184424091801, 0.000257491498217342,
0.00015177788709627, 0.000140234807389173, 0.000306101519148454,
0.000017179165166345, 0.000135170434011619, 0.000205417840695356,
0.00016662359565348,
                0.000024850296956713, 0.000200857250831839)
  )

# Prepare a data frame to store the results of Equation 44
Uterus_daily_AA_composition <- data.frame(day = days)
```

```
#Equation 44 calculated iterative for each essential amino acid
for (i in 1:nrow(Uterus_coefficients)) {
  #Coefficients needed to calculate Eq. 38 for each essential AA
  amino_acid <- Uterus_coefficients$AminoAcid[i]
  intercept <- Uterus_coefficients$Intercept[i]
  linear <- Uterus_coefficients$Linear[i]
  quadratic <- Uterus_coefficients$Quadratic[i]

  # Calculate the quadratic function representing the each essential AA (Eq.
38) for Uterus
  Eq_38_uterus <- intercept + linear * Uterus_daily_AA_composition$day +
quadratic * Uterus_daily_AA_composition$day^2

  # Equation 44
  Uterus_daily_AA_composition[[amino_acid]] <- Uterus_CP_daily * litter_size *
Eq_38_uterus/100
}

# Display the calculated values for all essential AAs by day of gestation
head(Uterus_daily_AA_composition)
```

3.5. Daily AA deposition calculated for fetus

```
#Coefficients for constructing quadratic functions representing essential AA
concentrations in the fetus (Table 4; columns 1, 8, 9, and 10)
fetus_coefficients <- data.frame(
  AminoAcid = c("Lys", "Arg", "His", "Ile", "Leu", "Met", "Cys", "Phe", "Thr",
"Trp", "Val"),
  Intercept = c(8.16490129205519, 5.90045508790045, 4.05952826990681,
3.63428883730943, 11.3838935982012,
                1.80131082158502, 2.33065309534037, 6.09945384128659,
4.67741019135327, 1.54349495527182, 7.70874271161135),
  Linear = c(-0.0317583685392876, 0.00290607570989544, -0.0313140853340223, -
0.00512895570020212, -0.0791511788778684,
             -0.00464059831345035, -0.0252824465398076, -0.0430895999326978, -
0.0159123394733633, -0.0124423634877261, -0.0561363596543605),
  Quadratic = c(0.0000717225356432773, 0.0000537252383908107,
0.000127257761536896, -0.0000002704396223446, 0.000333249761234987,
                0.0000231379152929724, 0.000148045654777025,
0.000179920888600822, 0.0000499611270666927, 0.0000474203142845735,
0.000243060963159327)
)

# Prepare a data frame to store the results of Equation 45
Fetus_daily_AA_composition <- data.frame(day = days)

#Equation 45 calculated iterative for each essential amino acid
for (i in 1:nrow(fetus_coefficients)) {
  #Coefficients needed to calculate Eq. 38 for each essential AA
  amino_acid <- fetus_coefficients$AminoAcid[i]
  intercept <- fetus_coefficients$Intercept[i]
  linear <- fetus_coefficients$Linear[i]
  quadratic <- fetus_coefficients$Quadratic[i]

  #Calculate the quadratic function representing the each essential AA (Eq.
38) for fetus
  Eq_38_fetus <- intercept + linear * Fetus_daily_AA_composition$day +
quadratic * Fetus_daily_AA_composition$day^2

  #Equation 45
  Fetus_daily_AA_composition[[amino_acid]] <- Fetus_CP_daily * litter_size *
(piglet_bw/1.46023)* Eq_38_fetus/100
}

# Display the calculated values for all essential AAs by day of gestation
head(Fetus_daily_AA_composition)
```

3.6. Daily AA deposition calculated for mammary glands

```r
#Essential AA concentration needed for Equation 46
Mammary_AA <- data.frame(
  Lys = 7.44,
  Arg = 6.23,
  His = 2.47,
  Ile = 4.02,
  Leu = 8.24,
  Met = 1.97,
  Cys = 1.57,
  Phe = 4.33,
  Thr = 4.29,
  Trp = 1.20,
  Val = 5.59
)

# Create a data frame to store results from Equation 46
Mammary_daily_AA_composition<-data.frame(day=seq(1,114,1))

#Equation 46 (Daily essential AA composition stored in the Mammary gland)
for (AA in names(Mammary_AA)) {
  Mammary_daily_AA_composition[[AA]] <- Mammary_gland_CP_daily * teats *
(Mammary_AA[[AA]] / 100)
}

# Display the calculated values for all essential AAs by day of gestation
head(Mammary_daily_AA_composition)
```

3.7. Daily AA deposition calculated for maternal body

```r
#Essential AA concentration needed for Equation 47
Maternal_AA <- data.frame(
  Lys = 7.4,
  Arg = 5.9,
  His = 3.2,
  Ile = 3.9,
  Leu = 7.2,
  Met = 1.8,
  Cys = 1.3,
  Phe = 4.0,
  Thr = 3.7,
  Trp = 1.3,
  Val = 5.0
)

# Create a data frame to store results from Equation 47
Maternal_daily_AA_composition<-data.frame(day=seq(1,114,1))

#Equation 47 (Daily essential AA composition stored in the maternal body)
for (AA in names(Maternal_AA)) {
  Maternal_daily_AA_composition[[AA]] <- Maternal_CP_daily *
(Maternal_AA[[AA]] / 100)
}

# Display the calculated values for all essential AAs by day of gestation
head(Maternal_daily_AA_composition)
```

3.8. Calculating and vizualizing the daily whole-body AA deposition model

```r
# Trim all data frames to 114 rows
Allantoic_daily_AA_composition <- Allantoic_daily_AA_composition[1:114, ]
Amniotic_daily_AA_composition <- Amniotic_daily_AA_composition[1:114, ]
Placenta_daily_AA_composition <- Placenta_daily_AA_composition[1:114, ]
Uterus_daily_AA_composition <- Uterus_daily_AA_composition[1:114, ]
Fetus_daily_AA_composition <- Fetus_daily_AA_composition[1:114, ]
Mammary_daily_AA_composition <- Mammary_daily_AA_composition[1:114, ]
Maternal_daily_AA_composition <- Maternal_daily_AA_composition[1:114, ]

#Equation 48
```

```r
All_tissues<-Allantoic_daily_AA_composition+
  Amniotic_daily_AA_composition+
  Placenta_daily_AA_composition+
  Uterus_daily_AA_composition+
  Fetus_daily_AA_composition+
  Mammary_daily_AA_composition+
  Maternal_daily_AA_composition

All_tissues$day<-seq(1,114,1)

All_tissues_long<- melt(All_tissues, id.vars = "day", variable.name = "AA",
value.name = "value")

# The 'end_points' are used to position the amino acid names as labels at the
end of the lines in the plot.
end_points <- All_tissues_long %>%
  group_by(AA) %>%
  filter(day == max(day))

# Plot with annotations
AA_plot <- ggplot(All_tissues_long, aes(x=day, y=value, color=AA)) +
  geom_line(size=1) +
  ylim(0, 17) +
  scale_y_continuous(breaks = seq(0, 20, by = 2)) +
  ylab("Amino acid deposition, g/d")+
  xlab("Day of gestation")+
  #theme_classic() +
  theme_bw()+
  geom_text(data = end_points, aes(x=day+2,label = AA), hjust = -0.2, vjust =
0,size=3)+
  theme(legend.position = "none")

ggplotly(AA_plot)
```
